# Seatbelts and road traffic collision injuries

**DOI:** 10.1186/1749-7922-6-18

**Published:** 2011-05-28

**Authors:** Alaa K Abbas, Ashraf F Hefny, Fikri M Abu-Zidan

**Affiliations:** 1 Trauma Group, Department of Surgery, Faculty of Medicine and Health Sciences, UAE University, Al-Ain

**Keywords:** Biomechanism, Road Traffic Collision, Injury, Mortality, Seatbelt compliance

## Abstract

Modification of seatbelts and their legislation played an important role in reducing morbidity and mortality of occupants in road traffic collisions. We aimed to review seatbelt development, its mechanism of action and its effects. Seatbelts reduce injury by preventing the occupant from hitting the interior parts of the vehicle or being ejected from the car. We have made a linear regression correlation between the overall seatbelt compliance and road traffic death rates in 46 high income countries to study the relationship between seatbelt use and mortality. There was a very highly significant negative correlation between the seatbelt compliance and road traffic death rates (R = - 0.77, F = 65.5, p < 0.00001). Seatbelt-related injuries include spinal, abdominal or pelvic injuries. The presence of a seatbelt sign must raise the suspicion of an intra-abdominal injury. These injuries can be reduced if seatbelts were applied correctly. Although seatbelts were recognized as an important safety measure, it still remains underused in many countries. Enforcement of seatbelt usage by law is mandatory so as to reduce the toll of death of road traffic collisions.

## Introduction

Road Traffic Collisions (RTC) are a leading cause of death, killing yearly more than 1.2 million worldwide, half of them between the age of 15 and 44. They cause further disabilities for more than 50 million injured patients [1]. RTC are often preventable. A reduction in the fatality rates can be achieved by improving vehicle crash safety and roadway design. The most important motor vehicle crash safety innovation which contributed to reduction in mortality has been the installation and proper use of seatbelts [2,3].

Some physicians in USA in the 1930s equipped their own cars with lap belts pushing the manufacturers to include them in the vehicle design [4]. This was not obligatory till 1964 when many USA states made it compulsory. Studies on seatbelts, as early as 1960, concluded that seatbelts reduce major fatal injuries [5]. Seatbelts were designed to prevent injury to the restrained passengers during RTC by preventing the occupant from hitting the vehicle components or being ejected from the vehicle [6].

Seatbelts reduce morbidity and mortality [5]. 50 - 80% of all deaths of RTC could have been prevented by properly used seatbelt [3,7]. Restrained occupants who have survived were shown to have more incidence of vertebral and intra-abdominal injuries compared with unbelted occupants [8]. It is not clear whether these injuries were caused by the seatbelts or they have been detected more in those who survived. Seatbelt effectiveness is related to the driver's behaviour and education level [9]. Incorrectly used seatbelts may cause fatal injuries [10]. Herby, we review the literature on seatbelts and their role in reducing road traffic collision injuries.

## Biomechanics and role of seat belts in RTC

**Figure 1:**
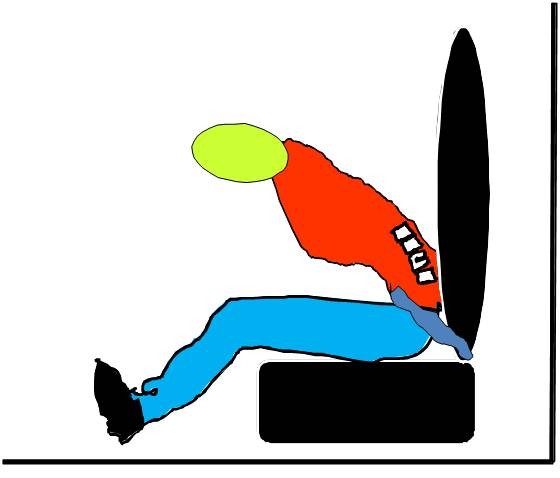
**Lap belts can be harmful**. They hold the body at two points and act as a fulcrum about which the body pivots causing major lumbar spine injuries.

Seatbelts reduce the severity of injury caused by RTC by restraining vehicle occupants in their seats and preventing them from hitting objects, or being ejected through the windows. They act to scatter the kinetic energy of the body which is released on rapid deceleration. This energy is disintegrated through the body skeleton [11]. Lap belts were used initially but many studies have shown that the lap belts are not sufficient as they hold the body at two points (Figure 1). The belt acts as a fulcrum about which the body pivots causing major force directed toward the lumbar spine [12]. They will not prevent head and chest from moving forward and hitting the windscreen or the steering wheel. Furthermore, the abdominal viscera may be injured.

Shoulder restraints were then introduced [5]. On 1968 the 3 point belt was made compulsory in UK. The emergency locking retractors were provided by Volvo on 1968. They lock the belt in sudden deceleration and prevent the body from bending forward [4].

When occupants are unrestrained in motor vehicle crashes, there will be three collisions. The first collision involves the vehicle and an external object, the second collision, which is responsible for most of the injuries, and can be prevented by seatbelt use, occurs between the unbelted occupant and the vehicle interior. The chest may hit the steering wheel and the head may hit the windscreen. Finally the third collision occurs when the internal organs of the body hit against the chest wall or the skeletal structure [3].

The amount of the energy and the direction of impact are major factors that determine the outcome of collisions. In front impact, there is deceleration of the vehicle as it hits another vehicle or a static object. Subsequently, the patient's lower extremities receive the initial energy impact which could result in different lower limb injuries including fracture dislocation of the ankle, femur fracture, knee dislocation, and posterior dislocation of the femoral head from the acetabulum as the pelvis override the femur. Furthermore, the head may hit the windscreen or the frame work around it [13-15]. Seatbelts will prevent the head from hitting the windscreen, chest from hitting the steering wheel, and the pelvis from overriding the femur.

A recent study has defined two types of frontal impacts; small overlap, where less than 30% of the vehicle front is involved in the crash, and large overlap where more than 30% is involved. Seatbelts were more effective in preventing serious head injuries in large overlap compared to small overlap frontal impacts [16].

In contrast, back impact leads to acceleration of the vehicle. This leads to hyperextension of the head (whiplash injury). This may lead to fractures of the posterior elements of the cervical spine including laminar, pedicle, and spinous process fractures. Seatbelts have a minor role on preventing such injuries but the head support will reduce it [13,17-20].

Side impact collision causes similar injuries as frontal impact. It also causes compression injuries to the pelvis which narrows its space. The head and neck can be tilted laterally causing nerve root avulsion and brachial plexus injury. Seatbelts have little effect on these injuries [17].

In rollover collisions, the unbelted passenger may hit any part of the interior of the passenger compartment. More severe injuries are seen because of the hard shaking motions of the passenger inside the vehicle during the rollover. The occupant can also be ejected from the vehicle, which increases the severity of injury. Seatbelts can prevent the occupant from being ejected from the car [17].

Unbelted occupants of RTC, become projectile within the vehicle which increases the risk of injury to other belted occupants. This effect will reduce the benefit of seatbelts in prevention of injury in belted patients as they become fixed targets for the projectile unbelted patients. To maximize the benefit of seatbelts, drivers, front seat passengers and back seat passengers should be all belted [21,22].

Seatbelt reduced perforating eye injuries by 60% [23]. Rear seat occupants are much safer than front seat occupants [24]. A study by Huelke and Compton [25] has shown that injury severity in restrained occupants was higher for front seat occupants compared with rear seat occupants. Rear seatbelt legislation was established in 1980s in USA, in 1986 in Sweden, in 1989 in New Zealand, and in 1993 in the European Union [26].

The relationship between velocity (V) and injury severity in belted occupants was studied, and showed a clear association between fatal injuries and high speed. This formula (Energy = 1/2 mass × V^2^), explains the relationship between the velocity of the vehicle and the amount of energy in RTC. Energy increases exponentially with increased velocity, so the more the velocity is the more serious and fatal the collision is. This relationship was also studied in a speed -injury curve. This curve shows clearly the strong relationship between high speed and severity of injury [27].

Incorrect seatbelt usage, which includes poor belt quality and poor adjustment in relation to the passenger's size, may cause serious intra-abdominal injuries. Fatal splenic injuries and splitting fractures of the third lumbar vertebra have been reported as a complication of incorrect application of the lap strap across the abdomen [10,12].

The combination of air bags and seat belts were added as a safety measure in the seventies and was made as a required safety measure for the car manufacturers in 1993. This combination has reduced the morbidity and mortality in motor vehicle collisions [28,29]. Drivers using airbags alone are 1.7 times more likely to suffer from cervical spine fracture, and 6.7 times more likely to suffer from spinal cord injury compared with those using both protective devices [8]. Maxillofacial and ocular injuries were reported as a complication of airbags when seatbelts are not used [30,31].

## Seatbelt-related injuries

Despite that seatbelts restrain the body to the car seat; the deceleration of the body may cause seatbelt-related injuries. The seatbelt sign is the bruising of the chest or abdominal wall with the diagonal or horizontal strap of the seatbelt [32,33]. The two point lap belts cause injuries to the abdomen, pelvis, and lumbar spine. With the 3 point restrains, the above injuries also occur with possible added injuries to the chest, heart, lung, brachial plexus and major vessels [34-36].

**Figure 2:**
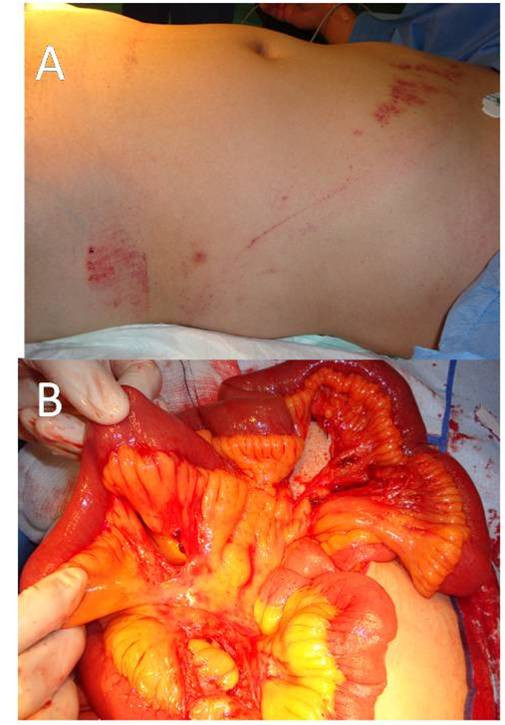
**A 30-year-old male driver with an abdominal seat belt sign (A) who had a laparotomy (B)**. The patient had abdominal tenderness and guarding. Abdominal CT scan has shown free intraperitoneal fluid without solid organ injury. Laparotomy has shown multiple mesenteric tears.

**Figure 3:**
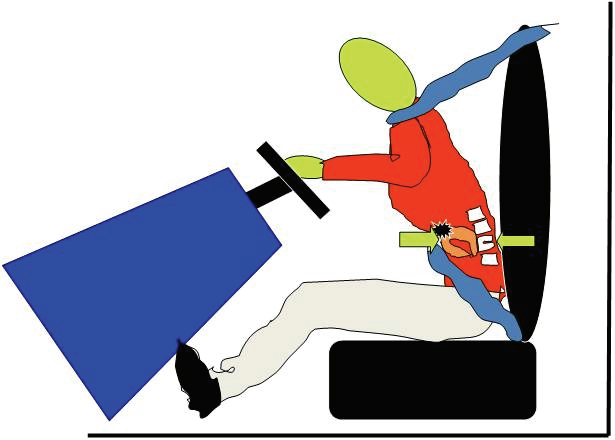
**Seatbelt syndrome is defined as a seatbelt sign associated with lumbar spine fracture and bowel perforation**.

Following a RTC, the presence of a seatbelt sign should raise the suspicion of an intra-abdominal injury [32,37,38] (Figure 2). In the presence of a seatbelt sign, the incidence of intestinal injury will increase. In a study of 117 RTC injured patients, 12% had seatbelt sign, of which 64% had abdominal injury. Those without seatbelt sign had fewer abdominal injuries (8.7%) [32,39,40]. Seatbelt syndrome is defined as a seatbelt sign associated with lumbar spine fracture and bowel perforation. (Figure 3) [12,33,36,41]. This is caused by hyperflexion of the spine around the lap strap in sudden deceleration leading to crushing of intra-abdominal contents between the spine and the seatbelt [13,42,43]. Fixed portions of the bowel such as proximal jejunum and distal ileum are more susceptible to injury than mobile portions. Mobile segments are more capable to escape the high pressure and resultant damage. Functional closed loops may sustain single or multiple blow-out perforations of the anti-mesenteric border of the gut due to raised intra-luminal pressure [44]. Similarly, esophagus and rectum may perforate with the same mechanism [45,46]. Intestinal strictures were reported as a seatbelt injury, where direct crush injury or contusion to the bowel wall can cause ischemia that ends in fibrosis. Strictures may involve more than one segment if the bowel was injured in more than one site [11,47]. "Chance fracture" which is a horizontal splitting of the vertebra that begins with the spinous process or lamina and extends anteriorly through the pedicles and vertebral body was first described by Chance GQ in 1948. This fracture has a strong relation with hollow viscus injury associated with lap belt injuries [48]. A seatbelt caused a chronic intermittent intestinal obstruction due to adhesions seven years following trauma [49]. Thoracic duct rupture and chylothorax as a complication of a seatbelt was reported after sudden increase in intra-abdominal pressure [50]. Similarly pancreatic transection at the neck may occur [51]. Intra-peritoneal rupture of distended urinary bladder may occur when the horizontal strap of the seatbelt increases the intra-vesical pressure [52]. Blunt traumatic aortic rupture [53], sternal fractures [41], clavicle fractures [32] and shoulder dislocations [54] were also reported as a complication of seatbelts. Cervical spinal injuries were noticed to be higher in restrained children than non-restrained children [19,32,55].

## Seatbelt compliance and road traffic collision deaths

**Figure 4:**
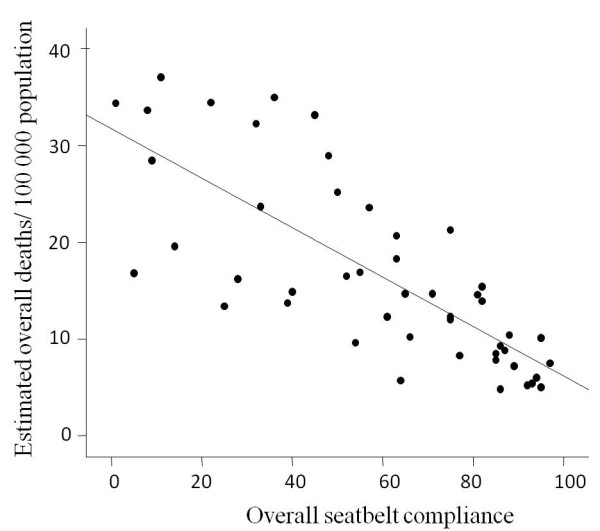
**Linear regression between the seatbelt compliance and road traffic death rates in 46 high-income countries**. The negative correlation was highly significant (R = - 0.77, F = 65.5, p < 0.00001).

We have studied the correlation between seatbelt use and road traffic deaths. A linear regression analysis was made between the overall seatbelt compliance and road traffic death rates in high income countries. Data for the high-income countries (defined as having a GNI $11 456 per capita or more) were retrieved from the WHO, road traffic injury prevention discussion paper (39 countries) [56]. More data were retrieved from MEDLINE, Google and Google scholar searching tools and data from another seven countries were added (Kuwait [57], New Zealand [58], Qatar [59], Saudia Arabia [11], Sweden [60], UAE [61], and USA [62]. We used data of high income countries which have overall seatbelt compliance for all occupants including the drivers, front seat passengers and back seat passengers. Data for estimated road traffic death rate per 100 000 populations for year 2007 were collected from the WHO road traffic injury prevention global status report on road safety [63]. The linear regression was done on data for 46 high-income countries. There was a very highly significant negative correlation between the seatbelt compliance and road traffic death rates (F = 65.5, p < 0.00001, R = - 0.77, Adjusted R square = 0.58) (Figure 4).

The above strong negative correlation between the seatbelt compliance and mortality rate can be explained by several factors. Unbelted occupants are more likely to be ejected from the vehicle after RTCs, with an increase of 70% in mortality compared with belted patients [62]. Unbelted occupants become projectile objects within the vehicle during RTCs which even increases the risk of injury of belted occupants who become a fixed target [21]. Furthermore, passengers comply less to seatbelts when they see the drivers not complying with seatbelts. Those carless drivers also take risky behavior like speeding, driving off the road, and disobeying the traffic law leading to fatal collisions [64].

Seatbelt usage has clearly reduced the mortality from road traffic collisions all over the world. Despite that, they remain underused [11,59,65]. It has been shown that gender may affect the compliance of seatbelt usage, but for all ages and seating positions, men had lower seatbelt wearing rates than women [66]. Males who were involved in crashes were three times more likely to be ejected from a car than females. Elder adults had higher rates of usage of seatbelts than teenagers [66-68].

Almost 60% of those killed in 2001 in vehicle crashes in USA didn't wear seatbelts [69]. Only 1% of the restrained passengers were ejected from car seats during a car crash. Of those ejected 73% were killed. In another study from North Carolina, the mortality rate was significantly higher in unbelted patients (7%) compared with belted patients (3.2%). Injury severity was higher in those unbelted patients [65].

*In summary*, seatbelts are considered as a defense line in preventing road traffic collision injury and death. It reduces injury by preventing the occupant from hitting the interior parts of the vehicle or being ejected from the car. Although seatbelts were recognized as an important safety measure, it still remains underused especially in developing countries. Seatbelt-related injuries can be reduced if seatbelts were applied correctly. The presence of a seatbelt sign must raise the suspicion of an intra abdominal injury. Several good practice interventions already tried and tested and can be implemented at low cost in most countries including strategies and measures that address some of the major risk factors for road traffic injuries. Setting laws' requiring seatbelts and child restrains for all occupants of the motor vehicles and, setting and enforcing speed limits and improving vehicle safety are essential. Enforcement of seatbelt usage is mandatory if we need to reduce the toll of death of road traffic collisions.

## Competing interests

The authors declare that they have no competing interests.

## Authors' contributions

AK participated in the literature review, data collection and preparation of the manuscript. AH helped in the idea and editing of the manuscript. FA participated in designing, preformed the statistical analysis, and critically revised the manuscript. All authors read and approved the final manuscript.
